# Pancreatic insulinoma misdiagnosed as atrial fibrillation: A case report from Iraq

**DOI:** 10.1097/MD.0000000000033456

**Published:** 2022-04-07

**Authors:** Aqeel Shakir Mahmood, Ahmed Dheyaa Al-Obaidi, Mazin Judy Ibrahim, Mustafa Najah Al-Obaidi, Ahmed A. Shakir, Reema Yousif Bakose, Naseem Wajdi, Hashim Talib Hashim

**Affiliations:** a Baghdad University, College of Medicine Baghdad, Baghdad, Iraq; b University of Baghdad, College of Medicine, Baghdad, Iraq; c F.I.B.M.S. Medical Oncology, ESMO Cert., Baghdad, Iraq; d General Surgery Department, Medical City Complex, Baghdad, Iraq; e Alza’Faranya General Hospital, Baghdad, Iraq.

**Keywords:** atrial fibrillation, hypoglycemia, insulinoma, misdiagnosis, tumor

## Abstract

Pancreatic insulinomas are the most frequent pancreatic endocrine neoplasms. They are insulin-secreting pancreatic tumors that induce extreme, recurrent, and near-fatal hypoglycemia. Insulinomas affect 1 to 4 individuals in a million of the general population and account for about 1% to 2% of all pancreatic tumors.

**Patient concerns:**

Recurrent episodes of sweating, tremor, weakness, confusion, palpitation, blurred vision, and fainting for 2 months and was misdiagnosed as having atrial fibrillation.

**Diagnosis:**

He was misdiagnosed as having atrial fibrillation to highlight the importance of atrial fibrillation as unusual mimicker of insulinoma and to encourage clinicians about the importance of early and appropriate management in such cases.

**Interventions:**

Endoscopic ultrasound for the pancreatic parenchyma was done, and it showed a hypoechoic homogenous mass located at the pancreatic head measuring 12 mm × 15 mm with no local vascular involvement, blue in elastography, hypervascular with Doppler study, and a normal pancreatic duct diameter.

**Outcomes:**

His condition was stable, and he was discharged home 2 days later.

**Conclusion:**

The diagnosis of insulinoma is usually difficult and late due to the extremely low incidence of the disease and the similarity of its clinical presentation to numerous other conditions, the most reported is epilepsy.

## 1. Introduction

Pancreatic insulinomas are the most frequent pancreatic endocrine neoplasms. They are insulin-secreting pancreatic tumors that induce extreme, recurrent and near-fatal hypoglycemia. Insulinomas affect 1 to 4 individuals in a million of the general population and account for about 1% to 2% of all pancreatic tumors. Both genders are equally at risk for developing insulinomas, regardless of their age. It has been observed that 90% of insulinomas are benign, 90% are localized lesions, 90% occur in the pancreas, and 90% are less than 2 centimeters in size.^[1]^ The majority are sporadic, while 10% are numerous and occur as part of multiple endocrine neoplasia syndrome type I. Insulinoma might be confused with other illnesses due to the similarity of its clinical presentation to numerous other conditions.^[2,3]^ Fasting hypoglycemia has often been thought to be the primary presenting characteristic of insulinoma. However, a few cases of insulinomas presenting only with postprandial hypoglycemia have been reported. According to a recent Mayo Clinic retrospective assessment, 6% of individuals with insulinoma reported having solely postprandial symptoms.^[4]^ We report this rare case of insulinoma being misdiagnosed as atrial fibrillation.

## 2. Case presentation

A 32-year-old male presented to Baghdad teaching hospital with recurrent episodes of sweating, tremor, weakness, confusion, palpitation, blurred vision, and fainting for 2 months duration. These episodes occurred around 15 times per month, at various times and in different places, although they were most noticeable in the morning. Each episode lasts about 20 minutes, were exacerbated by physical activity, and were alleviated by eating. He has a past medical history of atrial fibrillation of 2 years duration, and he is taking anti-arrhythmic and anticoagulant medications. The patient stated that he had gained around 17 kilograms in weight over the course of the preceding two months, going from weighing 61 kilograms to weighing 78 kilograms. The patient denied having chest pain, breathlessness, or episodes of seizure. Past surgical history was unremarkable. He went to a cardiology outpatient clinic, and the doctor had a suspicion that his condition was caused by his atrial fibrillation supported by electrocardiograph and the resembling symptoms. Therefore, he reassured him and modified his medication regimen.

**Figure 1.**
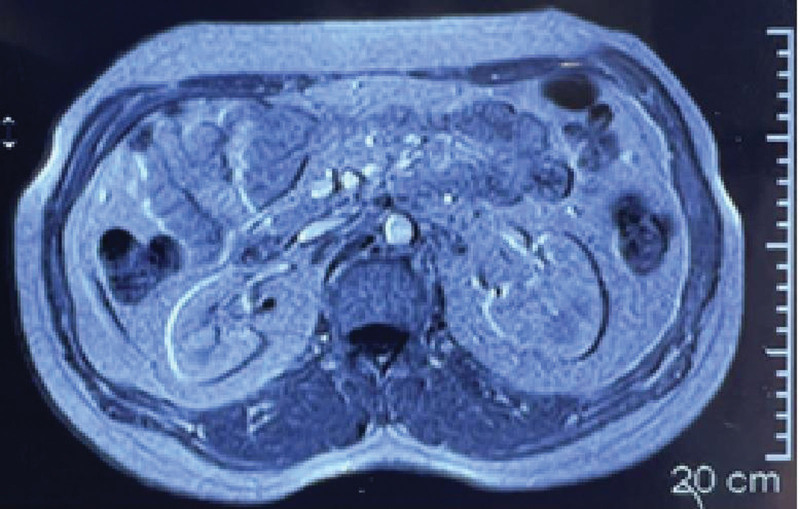
MRI of the abdomen with IV contrast showing a well-defined soft tissue mass that was round in shape, homogenously avidly enhanced, and measured about 11 × 13 mm at the head of the pancreas. MRI = magnetic resonance imaging.

**Figure 2.**
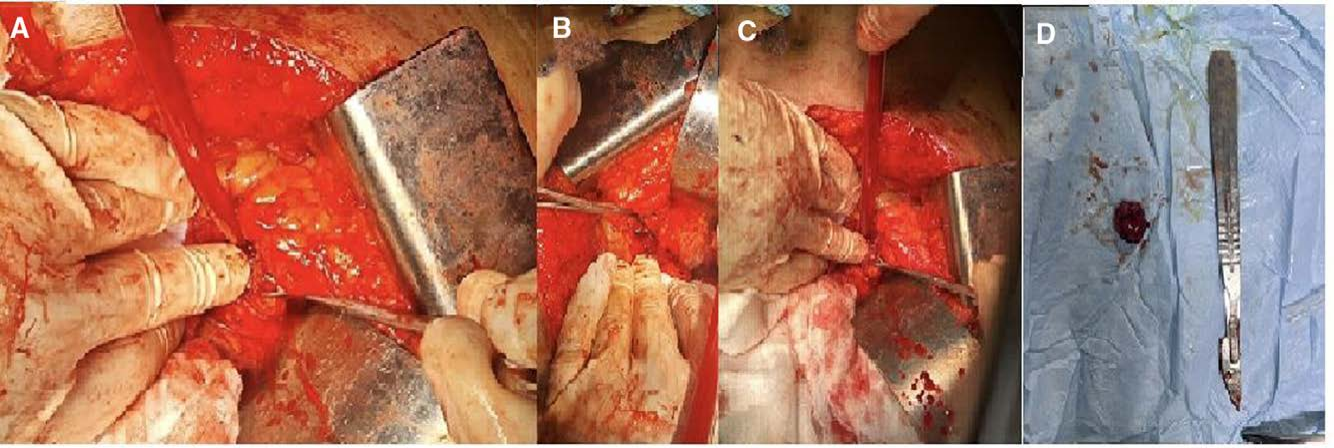
(A, B, C) Intra-operative images showing the tumor at the head of the pancreas. (D) This image showing the tumor after resection.

**Figure 3.**
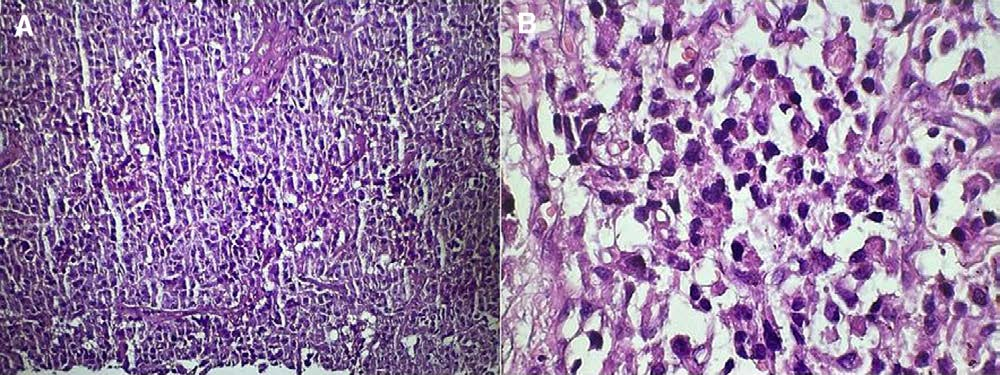
Histopathological slides demonstrating a diffuse infiltration of plasmacytoid epithelial cells under low power microscopy (A) and under high power microscopy (B).

**Figure 4.**
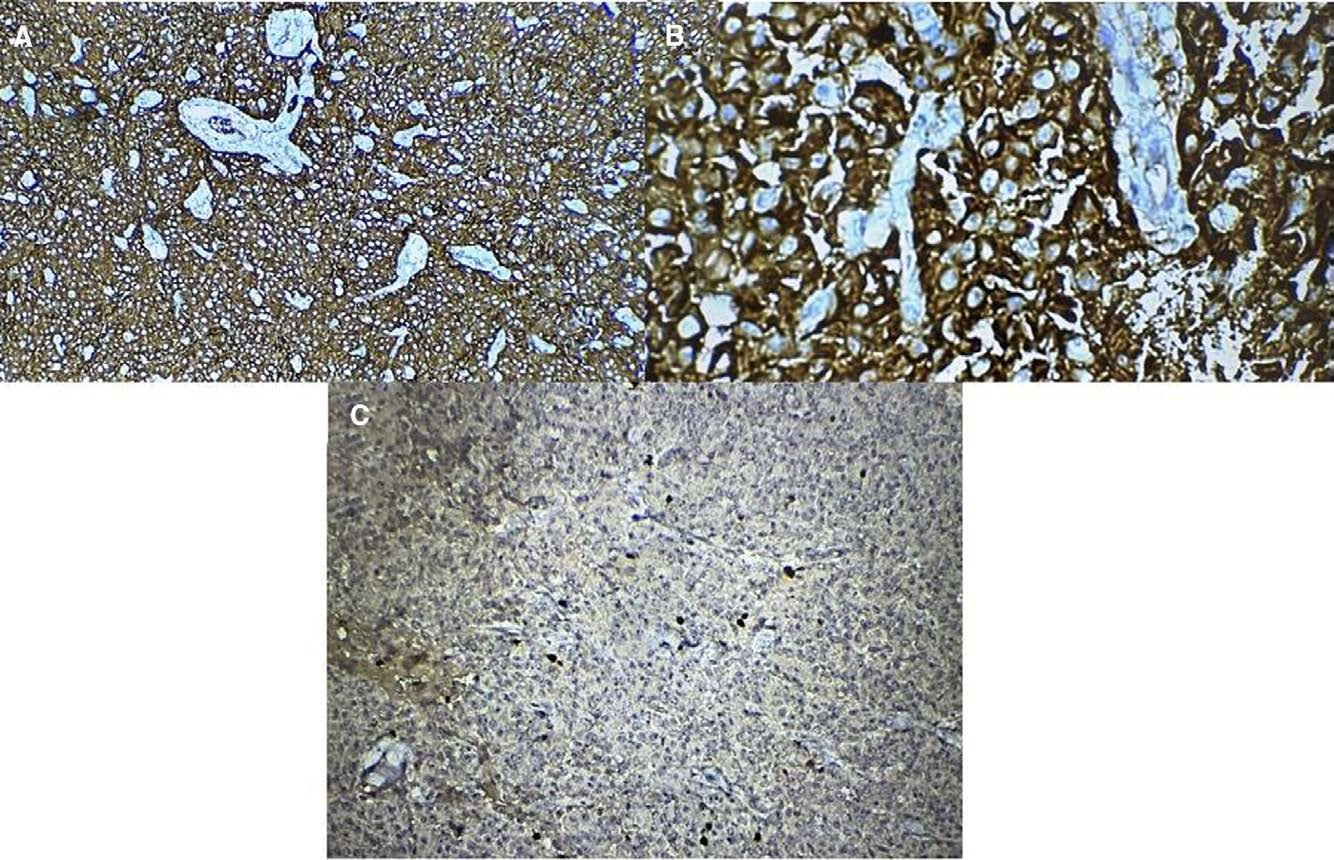
Immunohistochemistry with synaptophysin (A) and CD56 (B) showing diffusely strong positive results. (C) An immunohistochemistry with Ki67 and it showed a score of 3% of positively stained tumor cells.

On presentation, the patient was in a good clinical status, as his vital signs were all within normal ranges. On close inspection of the skin, there was a normal distribution of hair and no acanthosis nigricans, although there was acne on the face. The examination of other systems, including the cardiopulmonary examination, was unremarkable apart from irregular pulse. The lab investigations showed that the liver biochemical tests and renal function tests were both within normal levels. His pituitary-adrenal axis was intact. His electrocardiogram showed irregular QRS intervals with slow rate. The patient had hypoglycemia, as his blood sugar level was 43 mg/dL. The hypoglycemia was further evaluated using a 72-hour fasting test, and the patient was told to come immediately when experiencing abnormal symptoms. During the test, the patient developed symptoms similar to his episodic symptoms after approximately 18 hours of fasting. At the hospital’s emergency room, tests were done to check the patient’s blood glucose level, insulin level, circulating sulfonylurea levels, and insulin antibody levels. After these tests, the patient was given endogenous glucose, and his symptoms disappeared almost immediately. The results showed that he had hypoglycemia of 25 mg/dL, insulin level of 33.65 µU/mL, c-peptide level of 3.3 ng/mL, negative circulating sulfonylurea levels, and insulin antibody levels. The parameters were consistent with endogenous hyperinsulinism and suggestive of the presence of insulinoma. Accordingly, an magnetic resonance imaging (MRI) of the abdomen with IV contrast was done and showed a well-defined soft tissue mass that was round in shape, homogenously avidly enhanced, and measured about 11 × 13 mm at the head of the pancreas (as shown in the Fig. 1). The MRI also showed a normal pancreatic signal intensity and size with a normal pancreatic duct. The clinical, biochemical, and radiological findings were all consistant with the diagnosis of insulinoma. The MRI did not show any evidence of metastasis in the abdomen, as all other abdominal organs were normal. Endoscopic ultrasound for the pancreatic parenchyma was done, and it showed a hypoechoic homogenous mass located at the pancreatic head measuring 12 mm × 15 mm with no local vascular involvement, blue in elastography, hypervascular with Doppler study, and a normal pancreatic duct diameter. Finally, under endoscopic ultrasound guidance, a fine-needle aspiration was also taken. A CT scan of the chest, abdomen, and pelvis was done, and it did not show any evidence of metastasis. Ultrasound of the neck was performed, and no evidence of multiple endocrine neoplasia was seen. Brain MRI was negative for visible pituitary tumor. The patient was prepared for surgery, and due to the inadequate facilities, an open procedure was chosen instead of a laparoscopic one. During surgery, an incision was made in the upper abdomen, and the pancreas was revealed; however, the mass was not visible on inspection but was felt by palpation of the pancreatic head. Subsequently, an incision was made in the anterior pancreatic surface, and the mass was inoculated and ablated (as shown in Fig. 2). Postoperatively, the patient’s condition was stable, and he was discharged home 2 days later. A biopsy was taken with hematoxylin and eosin (H&E) staining and a histopathological slide showed a diffuse infiltration of plasmacytoid epithelial cells (as shown in Fig. 3). An immunohistochemistry with synaptophysin and CD56 was also done, and it showed diffusely strong positive results (as shown in Fig. 4A and B). An immunohistochemistry with Ki67 was done, and it showed an estimated score of 3% of positively stained tumor cells (as shown in Fig. 4C).

## 3. Discussion

Insulinomas are neuroendocrine, insulin-secreting tumors deriving mainly from islets cells of the pancreas, resulting in endogenous hyperinsulinism, which clinically manifests as blurred vision, palpitation, sweating, shivering, agitation to a greater extent, coma.^[5]^ Most of the insulinomas (90%) are benign and isolated tumors presenting at a median age of 50 years, except those associated with multiple endocrine neoplasia syndrome type I syndrome, which usually present earlier in the twenties and are multifocal.^[6]^ It usually presents with fasting hypoglycemia, although some cases also report post-prandial hypoglycemic symptoms. The diagnosis of insulinoma is confirmed by the presence of Whipple’s triad: clinical symptoms of hypoglycemia, biochemical evidence of endogenous insulin hypersecretion, and symptoms relief by the consumption of glucose.^[5,6]^

Biochemical investigations required in the diagnostic process include: insulin levels (normally less than 17 µU/mL), c-peptide level (normal value 0.78–1.89 ng/mL), blood sugar levels (normally 70–100 mg/dL), and circulating sulfonylurea levels (negative unless exogenous administration).^[7]^

A 48 to 72 hour fasting test is usually carried out, and once the patient experiences hypoglycemic symptoms, those biochemical tests are carried out, in which hypoglycemia, hyperinsulinemia, and elevated levels of c-peptide suggest insulinoma, while low levels of c-peptide and a positive circulating sulfonylurea indicate exogenous insulin administration.^[2,7]^

Preoperative and intraoperative localization of the tumor size are necessary to provide the best surgical plan and the best outcome. Preoperative localization is of high value, as surgeons previously relied on intraoperative palpation and ultrasound, which eventually led to a high conversion rate from laparoscopical to open resection of the tumor.^[8]^ Preoperative measures include trans-abdominal ultrasound, CT scan, and MRI with iv contrast, which have high sensitivity in insulinoma detection (100% for CT and MRI, and 90% for US). Another modality is endoscopic ultrasound, which is considered invasive, has limited availability, but has a high sensitivity of 90%, especially in detecting inulinomas of the head of the pancreas and, to a lesser extent, those of other pancreatic parts, and is used in cases of failure of localization by the other modalities. Another modality is pancreatic angiography with venous sampling after intra-arterial calcium stimulation, which can also be used in case the results of other modalities are equivocal or non-conclusive, as it is considered a highly invasive procedure.^[8,9]^

As most of the insulinomas are benign and solitary lesions, laproscopical resection of the affected pancreatic part is the treatment of choice, but an open procedure was decided for our patient due to inadequate facilities. However, certain difficulties might face surgeons during the course of the surgical plan, which is set according to preoperative findings, as certain insulinomas are embedded within the parenchymal tissue and others are not detectable by the preoperative measure and sometimes evenly by intraoperative palpation and ultrasound. In this case, the need for fine needle aspiration with an 18-gauge fine needle inserted into the suspicious lesion acting as a probe until the dome of the insulinoma is identified and a localized resection with serial measurement of insulin level intraoperatively is amenable.^[9,10]^

In case of multiple or malignant insulinomas, multiple enucleation is carried out as possible, often starting with liver metastatic lesions using an open approach rather than laparoscopic surgery, and the surgical options depend on the site and the extent of invasion, ranging from distal pancreatectomy with or without splenectomy to median pancreatectomy or the whipple procedure (pancreaticodedunectomy).^[10]^ In cases where tumors are irresectable or surgery fails with persistent symptoms, medical treatment can be used to counteract the effects of hyperinsulinism, such as octreotide, diazoxide, beta blockers, calcium channel blockers, and phenytoin.^[5]^

Pancreatic insulinomas are frequently misdiagnosed as a result of the presenting symptoms of hyperinsulinism: autonomic symptoms such as palpitation, tremor, and anxiety due to sympathetic stimulation; cholinergic symptoms such as diaphoresis, increased appetite, nausea, and salivation; and neuroglycopenic symptoms such as altered mental status, confusion, agitation, focal neurological signs, and even seizures and coma.^[6]^ The resemblance of those symptoms to other medical conditions, such as our case with atrial fibrillation, shows that rapid arrhythmias can present with the same autonomic and, to a lesser extent, neurological symptoms as fainting, which resulted in a late diagnosis of this patient’s insulinoma due to the rarity and co-existing AF.

Multiple case reports highlighted the frequently occurring incorrect misdiagnosis of insulinomas with epilepsy. A case was reported in Colombia about a middle-aged man with neurological symptoms of altered mental status and focal neurological signs resembling seizure, who was treated with anti-epileptic drugs with no remarkable improvement of his symptoms.^[11]^ Another case report stated that the patient was misdiagnosed as having epilepsy for a period of 8 years,^[12]^ and other cases were misdiagnosed as psychiatric illnesses such as double depression.^[13]^

## 4. Conclusion

The diagnosis of insulinoma is usually difficult and late due to the extremely low incidence of the disease and the similarity of its clinical presentation to numerous other conditions, the most reported is epilepsy. We report this rare case of pancreatic insulinoma being misdiagnosed as having atrial fibrillation in order to highlight the importance of atrial fibrillation as unusual mimicker of insulinoma and to encourage clinicians about the importance of early and appropriate management in such cases.

## Author contributions

**Conceptualization:** Hashim Talib Hashim.

**Data curation:** Aqeel Shakir Mahmood, Mustafa Najah Al-Obaidi, Ahmed A. Shakir, Naseem Wajdi, Hashim Talib Hashim.

**Funding acquisition:** Aqeel Shakir Mahmood.

**Investigation:** Aqeel Shakir Mahmood, Mazin Judy Ibrahim, Ahmed A. Shakir.

**Methodology:** Mazin Judy Ibrahim.

**Project administration:** Hashim Talib Hashim.

**Resources:** Aqeel Shakir Mahmood, Mazin Judy Ibrahim, Naseem Wajdi, Hashim Talib Hashim.

**Software:** Ahmed Dheyaa Al-Obaidi, Reema Yousif Bakose.

**Validation:** Ahmed Dheyaa Al-Obaidi, Mustafa Najah Al-Obaidi.

**Writing – original draft:** Ahmed Dheyaa Al-Obaidi, Mustafa Najah Al-Obaidi, Reema Yousif Bakose.

**Writing – review & editing:** Hashim Talib Hashim.
